# Unilateral Isolated Proximal Femoral Focal Deficiency

**DOI:** 10.1155/2013/637904

**Published:** 2013-07-28

**Authors:** Emek Doğer, Şule Y. Köpük, Yiğit Çakıroğlu, Özgür Çakır, Gülseren Yücesoy

**Affiliations:** ^1^Department of Obstetrics and Gynecology, School of Medicine, Kocaeli University, Umuttepe Campus, 41380 Uctepeler, Kocaeli, Turkey; ^2^Department of Radiology, School of Medicine, Kocaeli University, Umuttepe Campus, 41380 Uctepeler, Kocaeli, Turkey

## Abstract

*Objective*. To discuss a patient with a prenatal diagnosis of unilateral isolated femoral focal deficiency. *Case*. Antenatal diagnosis of unilateral isolated femoral focal deficiency was made at 20 weeks of gestation. The length of left femur was shorter than the right, and fetal femur length was below the fifth percentile. Proximal femoral focal deficiency was diagnosed. After delivery, the diagnosis was confirmed with skeletal radiographs and magnetic resonance imaging. In prenatal ultrasonographic examination, the early recognition and exclusion of skeletal dysplasias is important; moreover, treatment plans should be initiated, and valuable information should be provided to the family.

## 1. Introduction

Isolated femoral hypoplasia is a rare congenital limb anomaly with an incidence of 1.1–2 in 100 000 live births. Its prenatal diagnosis has increased with the widespread use of fetal ultrasonography [1]. Mental disorders and chromosomal abnormalities are not usually present with proximal focal femoral deficiency (PFFD). Surgical reconstruction results in a good prognosis [2]. However, some cases of femoral hypoplasia that include skeletal malformations may be accompanied by global dysplasia syndromes, and termination of pregnancy may be the only reasonable option in such cases [3]. An important issue is to determine whether isolated femoral abnormality is part of the syndrome. Armstrong et al. [4] indicated that although accurate diagnosis is only possible in 6 of 9 patients, termination of pregnancy is more commonly preferred in cases with femoral anomalies.

In our study, we report a case of isolated, unilateral PFFD detected at 20 weeks of gestation during prenatal ultrasonography.

## 2. Case

**Figure 1:**
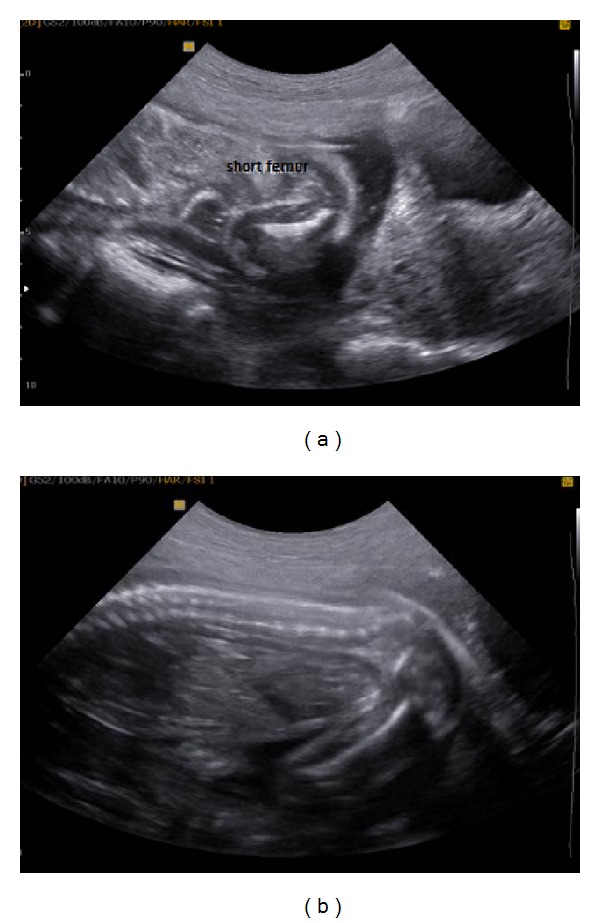
Ultrasound images of both the right and left femurs at 20 wg. Left femur: 19.7 mm and the right femur: 31.6 mm were measured.

A 27-year-old patient (G1P0) was referred for an ultrasound scan at 20 weeks of gestation. Ultrasonographic examination was performed with a 2–6 MHz abdominal ultrasound probe (SonoAce X8 ultrasound device; Samsung Medison Co., Seoul, South Korea). This examination revealed that the left femur of the fetus was considerably shorter than the right femur, and the length of the left femur was below the fifth percentile (19.7 mm versus 31.6 mm, resp.). The distal epiphyseal region appeared normal. The measurements of all other long bones (lengths, structures), head circumference, and abdominal circumference were normal according to gestational age. No other skeletal abnormalities; thorax anomalies; or cardiac, gastrointestinal, genitourinary, and neurological signs of fetal abnormalities were observed (Figure 1). The fetal facial profile was normal. The parents were nonconsanguineous and healthy. There was no family history of skeletal abnormalities or any other diseases. Moreover, there was no history of gestational diabetes, drug use, teratogen or radiation exposure, or a history of viral infection during the gestational period. The first trimester combined screening test results revealed a low risk for trisomy 21. Amniocentesis results indicated a normal karyotype. The presumptive diagnosis was PFFD. After a spontaneous rupture of membranes, a 2450 g male infant was delivered at the 33rd gestational week, with an Apgar score of 9-10.

**Figure 2:**
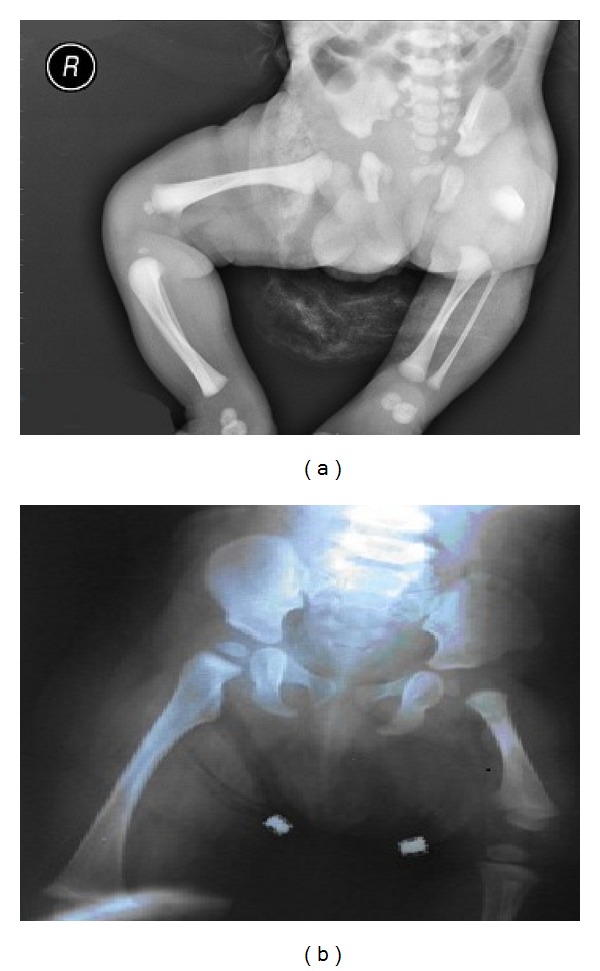
The first week after birth (a) and 18 months later (b) with plain radiography, comparative view of the left and right femurs. Normal right lower extremity and left femoral deficiency in (a). The shortening of left femur, femur head and neck in (b).

**Figure 3:**
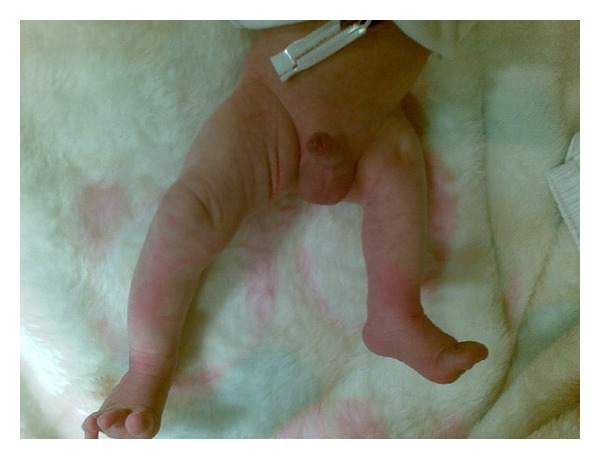
Postnatal appearance of the thigh. Short externally rotated thigh was shown in postnatal appearance of fetus.

X-ray scans, pelvic and extremity magnetic resonance imaging scans, and ultrasound examination confirmed the diagnosis of isolated, unilateral PFFD (Figures 2 and 3). The neonate's facial appearance was normal. No other abnormality was detected. Six months after delivery, abnormalities of the femoral head and femoral neck acetabulum were observed on ultrasonographic examination, and the measurements were lower than the expected percentile; therefore, according to the Aitken classification, a diagnosis of type A PFFD was confirmed. The child is currently 2 years old and has good mobility of the hip joint. Moreover, the postnatal followup has been uneventful.

## 3. Discussion

Congenital hypoplasia of the femur is a principal sign of 4 uncommon malformations: (a) PFFD, (b) femur/fibula/ulnar hypoplasia (FFU), (c) femoral hypoplasia/unusual facial syndrome (FH/UFS), and (d) limb/pelvis-hypoplasia/aplasia syndrome [1]. PFFD is a rare congenital disorder resulting from the failure of the development of the subtrochanteric portion of the femoral shaft that is characterized by shortness, deformity, and dysfunction [5]. The unilateral form is approximately 85–90% of all cases [6]. It is usually sporadic, although a few familial cases have been described; moreover, its genetic transmission mode is unknown [7]. The interval between the fourth and eighth weeks of gestation is the most critical period for skeletal development. Poor diabetic control in the early weeks of pregnancy, drug exposure (thalidomide), viral infections, radiation, focal ischemia, chemical toxicity, trauma, and causes of familial transmission are some of the etiologic factors [6, 8, 9].

**Figure 4:**
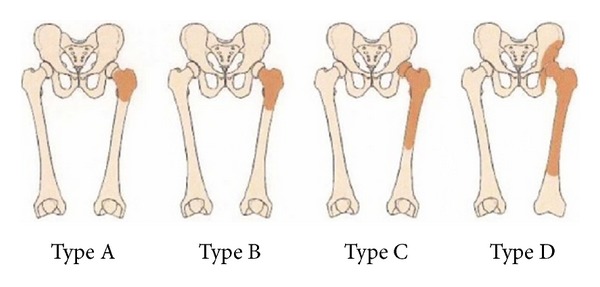
The Aitken classification of PFFD (with permission of Dr. Jeanty).

Various PFFD classifications have been made on the basis of the relationship between the proximal end of the femur and the acetabulum. The most commonly used classification is the one described by Aitken and modified by Amstutz [10, 11]. There are 4 classifications (A–D), according to the presence of the femoral head, a stable hip joint, or acetabular hypoplasia (Figure 4). The Aitken classification does not take into account the classification of cartilage and soft tissue abnormalities.

Prenatal diagnosis of syndromes associated with abnormalities of the femur is possible; however, although only 19% of cases have been diagnosed prenatally, 68% of cases have been diagnosed postnatally [4]. When a short femur is detected, a differential diagnosis should be made with kyphomelic dysplasia, campomelic dysplasia, osteogenesis imperfecta, achondroplasia, achondrogenesis, thanatophoric dysplasia, short limb polydactyly, and malformations of skeletal dysplasias (e.g., chondroectodermal). This disease also affects other long bones, and bilateral involvement as well as frontal, cranial abnormalities is also observed [12]. When these findings are not observed, other subgroup of femoral hypoplasia should be considered. As FH/UFS closely associated with diabetic embryopathy, bilateral femoral hypoplasia, short nose, long philtrum, thin upper lip, small lower jaw, cleft palate is characterized by containing the facial dimorphism [13]. For differential diagnosis of this disease, two- or three-dimensional ultrasonographic examinations are required to confirm that the fetal facial profile is normal. Femur, fibula, and ulnar bone defects and those various combinations were observed in femur/fibula/ulnar complex. However, all extremities and many pelvic deformities are affected in autosomal recessive inherited Limb/pelvis-hypoplasia/aplasia syndrome [1]. If the fetal face and all the other long bones are normal and the bone mineralization is normal, PFFD can be confirmed. In all, 30–60% of anomalies are associated with PFFD; these include fibular hemimelia; pes equinovarus; and rarely include oligodactyly, tibia bone bending, the absence of the knee cross ligaments, spinal dysraphism, and microcephaly [8, 14–16].

The diagnosis of femoral hypoplasia is possible after the second trimester of pregnancy [17–20]. Some cases diagnosed at approximately 14 weeks of gestation by transvaginal ultrasonography have been reported in the literature [5]. During ultrasonographic examination, length discrepancies and disproportion between femurs and other bones are the diagnostic determiners of PFFD. Other long bone deficiencies or hypoplasia is very rare. If these are observed, usually a short fibula is associated with a short femur. Whenever short femur is detected, the femoral head, length of hemipelvis, and fibular hypoplasia should be examined carefully. Acetabular malformations are difficult to recognize in the prenatal period and may not be detected by ultrasound. Helical computed tomography can be performed, and an accurate three-dimensional diagnosis can be made; however, this will expose the fetus to radiation in utero [20]. After birth, although skeletal X-ray scans are useful for diagnosis, the condition may be accurately classified after 1 or 2 years [21]. Early neonatal ultrasound and magnetic resonance imaging are useful for the classification of PFFD [22]. When the difference between femoral lengths is not obscure in the prenatal period, diagnosis may be delayed until the child begins to walk. 

Patients with simple femoral hypoplasia do not usually develop a secondary deformity, and the problem is limited to asymmetric legs. If not corrected, PFFD results in an unpleasant appearance, excessive energy consumption during walking, scoliosis causing back pain, and related symptoms. Surgical correction is required for significant shortness. In PFFD type A, minimal side effects are observed, whereas types B, C, and D require surgical correction. Valgus osteotomy, arthrodesis of the knee, distal femoral epiphysiodesis, Van Nes rotationplasty, the Syme amputation, and femoral lengthening operations are the most commonly used procedures [8, 23]. The aim of these surgical procedures is to synchronize the length of the leg, stabilize the feet, and increase the pelvofemoral stability. After orthopedic correction, the long-term prognosis is usually good [8, 23, 24].

PFFD is not associated with chromosomal abnormalities, and patients have normal intelligence. The literature contains case reports in which termination of pregnancy has been selected before fetal viability [13, 18]. In prenatal ultrasonographic examination, the early recognition and exclusion of skeletal dysplasias should be aimed; moreover, treatment plans should be initiated, and valuable information should be provided to the family.

## References

[1] Camera G, Dodero D, Parodi M, Zucchinetti P (1993). Antenatal ultrasonographic diagnosis of a proximal femoral focal deficiency. *Journal of Clinical Ultrasound*.

[2] Bevan-Thomas WH, Millar EA (1967). A review of proximal focal femoral deficiencies. *Journal of Bone and Joint Surgery A*.

[3] Parilla BV, Leeth EA, Kambich MP, Chilis P, MacGregor SN (2003). Antenatal detection of skeletal dysplasias. *Journal of Ultrasound in Medicine*.

[4] Armstrong R, McCann E, Fryer A, Bricker L, Platt MJ (2009). A review of prenatally detected femoral abnormalities. *Clinical Dysmorphology*.

[5] Bronstein M, Deutsch M (1992). Early diagnosis of proximal femoral deficiency. *Gynecologic and Obstetric Investigation*.

[6] Gonçalves LF, De Lucca GR, Vitorello DA (1996). Prenatal diagnosis of bilateral proximal femoral hypoplasia. *Ultrasound in Obstetrics and Gynecology*.

[7] Makino Y, Inoue T, Shirota K, Kubota S, Kobayashi H, Kawarabayashi T (1998). A case of congenital familial short femur diagnosed prenatally. *Fetal Diagnosis and Therapy*.

[8] Jeanty P, Kleinman G (1989). Proximal femoral focal deficiency. *Journal of Ultrasound in Medicine*.

[9] Hadi HA, Wade A (1993). Prenatal diagnosis of unilateral proximal femoral focal deficiency in diabetic pregnancy: a case report. *American Journal of Perinatology*.

[10] Aitken GT, Aitken GT (1969). Proximal femoral focal deficiency: definition, classification and management. *Proximal Femoral Deficiency: A Congenital Anomaly*.

[11] Amstutz HC, Aitken GT (1969). The morphology, natural history and treatment of proximal femoral deficiency. *Proximal Femoral Focal Deficiency: A Congenital Anomaly*.

[12] Alanay Y, Krakow D, Rimoin DL, Lachman RS (2007). Angulated femurs and the skeletal dysplasias: experience of the International Skeletal Dysplasia Registry (1988–2006). *American Journal of Medical Genetics A*.

[13] Paladini D, Maruotti GM, Sglavo G (2007). Diagnosis of femoral hypoplasia-unusual facies syndrome in the fetus. *Ultrasound in Obstetrics and Gynecology*.

[14] Filly AL, Robnett-Filly B, Filly RA (2004). Syndromes with focal femoral deficiency: strengths and weaknesses of prenatal sonography. *Journal of Ultrasound in Medicine*.

[15] Johansson E, Aparisi T (1983). Missing cruciate ligament in congenital short femur. *Journal of Bone and Joint Surgery A*.

[16] Sirota L, Bar-Ziv J, Landman J, Dulitzky F (1987). Proximal femoral focal deficiency associated with severe brain atrophy. *Israel Journal of Medical Sciences*.

[17] Cuillier F, Cartault F, Moreau ML, Lemaire P (2005). Antenatal presentation of isolated femoral hypoplasia discovered at 18 weeks of gestation. *Fetal Diagnosis and Therapy*.

[18] Oh KY, Frias AE, Byrne JLB, Kennedy AM (2008). Unilateral short femur—what does this mean? Report of 3 cases. *Ultrasound Quarterly*.

[19] Mailath-Pokorny M, Timor-Tritsch IE, Monteagudo A, Mittal K, Konno F, Santos R (2011). Prenatal diagnosis of unilateral proximal femoral focal deficiency at 19 weeks’ gestation: case report and review of the literature. *Ultrasound in Obstetrics and Gynecology*.

[20] Otera Y, Morokuma S, Yumoto Y (2009). Prenatal three-dimensional images of proximal focal femoral deficiency produced by helical computed tomography. *Fetal Diagnosis and Therapy*.

[21] Koman LA, Meyer LC, Warren FH (1982). Proximal femoral focal deficiency: natural history and treatment. *Clinical Orthopaedics and Related Research*.

[22] Hillmann JS, Mesgarzadeh M, Revesz G, Bonakdarpour A, Clancy M, Betz RR (1987). Proximal femoral focal deficiency: radiologic analysis of 49 cases. *Radiology*.

[23] Gillespie R, Torode IP (1983). Classification and management of congenital abnormalities of the femur. *Journal of Bone and Joint Surgery B*.

[24] Westberry DE, Davids JR (2009). Proximal focal femoral deficiency (PFFD): management options and controversies. *HIP International*.

